# Generation mechanism and prediction of an observed extreme rogue wave

**DOI:** 10.1038/s41598-022-05671-4

**Published:** 2022-02-02

**Authors:** Johannes Gemmrich, Leah Cicon

**Affiliations:** 1 Physics and Astronomy, University of Victoria, Victoria, BC Canada; 2 School of Earth and Ocean Sciences, University of Victoria, Victoria, BC Canada

**Keywords:** Natural hazards, Ocean sciences, Physical oceanography

## Abstract

Rogue waves are individual ocean surface waves with crest height $\eta$ or trough-to-crest height *H* that are large compared to the significant wave height $H_s$ of the underlying sea state: $H/H_s>2.2$ or $\eta /H_s>1.25$. The physics of rogue wave generation and the potential of predicting the rogue wave risk are open questions. Only a few rogue waves in high sea states have been observed directly, but they can pose a danger to marine operations, onshore and offshore structures, and beachgoers. Here we report on a 17.6m high rogue wave in coastal waters with $\eta /H_s=1.98$ and $H/H_s=2.9$ which are likely the largest normalized heights ever recorded. Simulations of random superposition of Stokes waves in intermediate water depth show good agreement with the observation. Non-linear wave modulational instability, a well known cause for rogue waves in laboratory settings, did not contribute significantly to the rogue wave generation. A parameter obtained from a routine spectral wave forecast provides a practical risk prediction for rogue waves. These results confirm that probabilistic prediction of oceanic rogue waves based on random superposition of steep waves are possible and should replace predictions based on modulational instability.

## Introduction

The observation of the “Draupner” wave ( $\eta /H_s = 1.55$, $H/H_s=2.15$, $H_s=11.9$ m) on 1 January 1995 proved that the existence of so-called rogue or freak waves are not a seafarer’s folklore^1^. Technically, rogue waves are waves in the tail of the wave height or crest height probability distribution, $H/H_s>2.2$ or $\eta /H_s>1.25$ where $H_s$ is the average of the highest one-third waves^2^. Thus, rogue waves occur in any background sea state. Not surprisingly, rogue waves in high sea states receive the most attention. In particular, the “Draupner” wave, the “Andrea” wave ($\eta /H_s = 1.62$, $H/H_s=2.30$, $H_s=9.2$ m), and the “Killard” wave ($\eta /H_s = 1.57$, $H/H_s=2.29$, $H_s=11.4$ m) are well studied^3–7^. Of main interest is the probability of encountering such a rogue wave, and which physical processes, if any, can alter the underlying probability density function for wave or crest heights^2,8–10^. In linear theory, the sea is a random superposition of linear waves of a narrow frequency spectrum and the individual wave heights *H* follow the Rayleigh distribution^11^. The probability of a wave or crest exceeding the significant wave height by a multiple *z* is

1 $$\begin{aligned} P(H/H_s>z)=e^{-2z^2},\text { and}\,\, P(\eta /H_s>z)=e^{-8z^2} \end{aligned}$$

for wave heights and crest heights, respectively. Thus, a rogue wave with $H/H_s>2.2$ is expected with a probability 1/16,000, or since the dominant wave period in the ocean is $O(10\,s)$ a rogue wave is expected about every two days. Allowing for finite amplitudes, wave crests become taller and troughs shallower, due to the presence of bound higher harmonics^12^. At second order this Stokes correction affects crest height distributions, and exceedance probabilities corrected for second order have become the standard model for estimating extreme crest height probabilities^13–15^. For typical wave steepness the probability of rogue crests increases by a factor 5 compared to small-amplitude linear theory. For narrow spectral bandwidth, wave height distributions are not affected by second order corrections. Observations from the GORM platform in the North Sea showed that the distribution of moderate wave heights $H/H_s\lesssim 2$ is consistent with second order models accounting for finite spectral bandwidth, but extreme waves $H/H_s\gg 2$ seem to follow a different distribution^9^. Most observed wave height distributions are based on relatively short records and the extreme values are limited to moderate normalized wave heights where the distributions are consistent with second order models^16^. Using synthesized surface elevation it was shown that the increased probabilities of extreme waves, as observed in the GORM data set, cannot be explained by second order alone, but is consistent with fourth order Stokes theory of bound waves^8^.

Rogue waves can be the consequence of third order nonlinear four-wave interactions between free waves forming a modulational instability^17^ where individual waves can grow as sidebands, drawing energy from neighbouring waves. For long-crested waves this mechanisms is well documented in wave flumes^18–20^ and it is the basis for the Benjamin–Feir Index (BFI) which is routinely being used as rogue wave risk predictor^21^. Under modulational instability the tails of the probability density function of wave crests and wave heights will be higher than predicted by second order theory. However, as waves become more directionally spread modulational instability becomes less effective, as has been shown in wave tank experiments^22^ and numerical simulations^23,24^, and for observed real ocean rogue waves BFI has little predictive power^25^.

Here we report observations of an extreme rogue wave ($\eta /H_s=1.98$, $H/H_s=2.91$, $H_s=6.05\,$ m) off Vancouver Island, BC, Canada. According to second order theory^13^ such an event ($\eta /H_s=1.98$, wave steepness parameter $R=0.23$) would occur once in 1300 years. The corresponding Benjamin-Feir Index $BFI=0.13$ indicates that modulational instability did not play a role in generating such an extreme wave. However, Monte-Carlo simulation of random superposition of fourth order Stokes waves^8^ drawn from the observed wave energy spectra yield wave and crest height distributions in good agreement with the 10 month buoy record, including the extreme rogue wave. Furthermore, we show that the crest-trough correlation $r\,$^25–27^ calculated from a WAVEWATCH III^®^^28^ (WW3) wave model has strong predictive power for rogue wave risk prediction from a standard wave forecast model.

## Rogue wave observation

**Figure 1:**
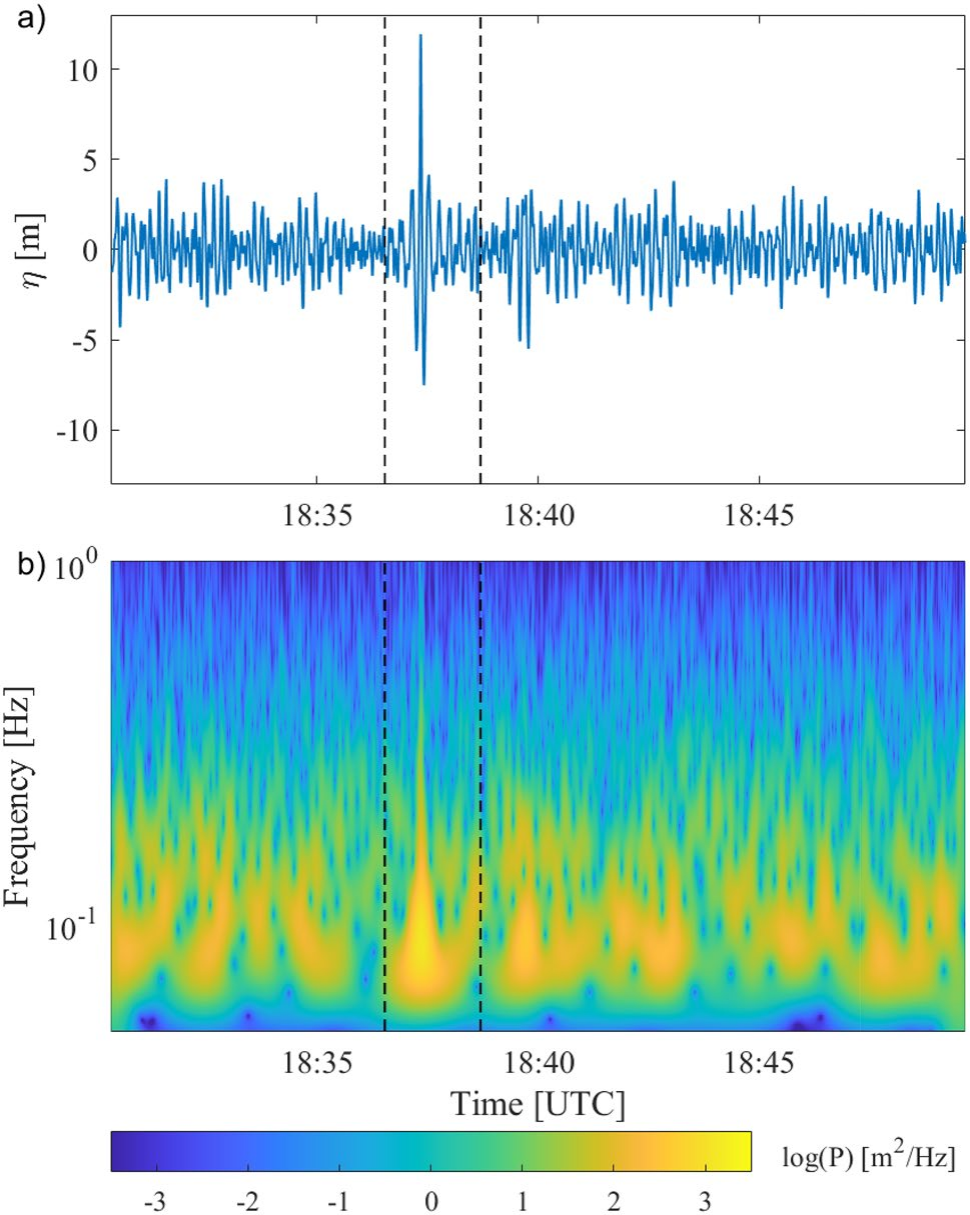
Rogue wave recorded on Nov 17, 2020. Vertical dashed lines indicate the wave group containing the rogue wave. (**a**) Surface elevation $\eta$. (**b**) Spectrogram of surface elevation using the Morlet wavelet.

A 0.9 m CoastScout wave buoy (MarineLabs) was deployed 13/08/2020 at Amphitrite Bank (48.9$^{\circ }$ N, 125.6$^{\circ }$ W) about 7 km offshore in 45 m water depth. The buoy records surface elevation at 5 Hz from a 3-D inertial measurement unit (IMU), and from differential GPS. Data are recorded in 20 min bursts every 30 min and are available in near-real time. Here we analyze its data record until 21/05/2021, covering a wide range of sea states 0.5 m < $H_s$ $\le$ 10.1 m. The record includes an extreme rogue wave on 17/11/2020 (Fig. 1a) with $\eta =11.96$ m crest height (recorded by IMU and GPS sensor) when the significant wave height was $H_s=6.05$ m. The total wave height *H* was 17.6 m (19.5 m) based on the preceding (following) trough. The significant wave height had increased rapidly from a minimum $H_s=1.93\,$ m 18 h prior to the event. The rogue wave is the fourth crest in a group of about 10 waves. The crests of the preceeding and following waves were much smaller than the rogue wave crest, with $\eta =3.27\,$ m and $\eta = 4.13\,$ m, respectively. This is consistent with the fact that rogue waves generally occur near the centre of a group and are unexpected, i.e. there is not a gradual build-up of individual wave heights^29,30^.

**Figure 2:**
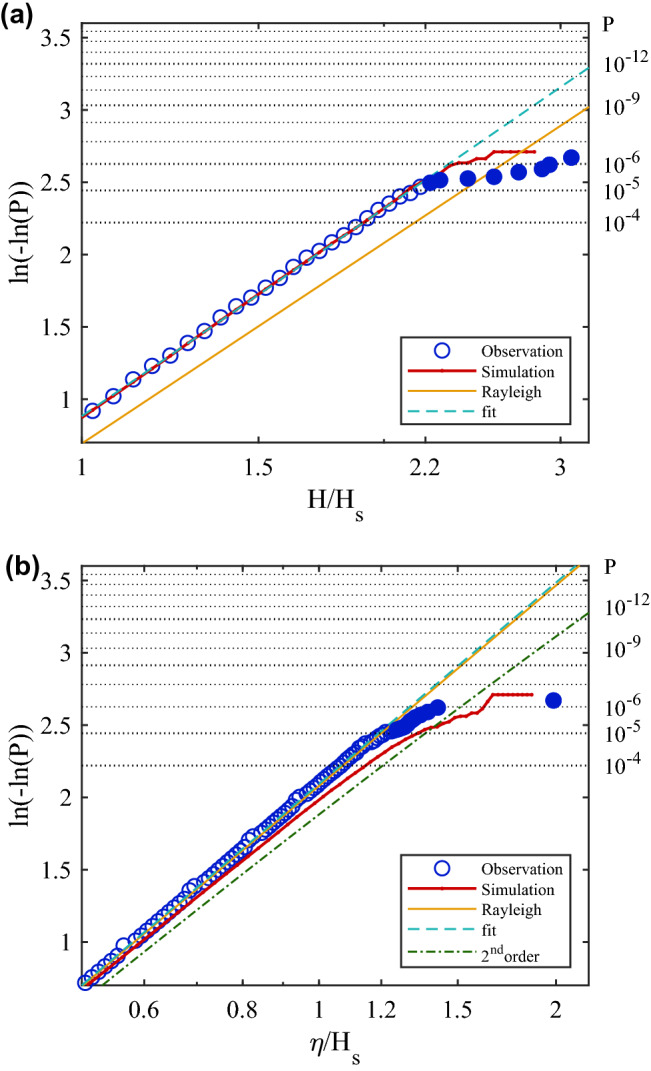
Exceedance probability P of (**a**) non-dimensional wave height $H/H_s$, and (**b**) non-dimensional crest height $\eta /H_s$. Second order curve is based on steepness $R=0.23$^13^. Filled symbols show observed rogue waves.

The spectrogram (Fig.1b) reveals bursts of energy associated with wave groups and a dominant frequency $f_p=0.078\text { Hz}$ (corresponding to a dominant period $T_p=12.8 \text { s}$), and a second peak at $f_2=0.156 \text { Hz}$ ($T_2=6.4\text { s}$). The group containing the rogue wave has the highest energy and during the rogue wave the energy is spread continuously over a wide frequency range from $f=0.06\text { Hz}$ to $f=0.9\text { Hz}$. This implies that the rogue wave is the combination of energetic waves from a broad range of scales. As the group evolves the high frequency energy rapidly decreases and the low frequency peak exhibits a continuous upshift and individual wave heights decrease. This spectral shape is unique to the wave group containing the rogue wave.

**Figure 3:**
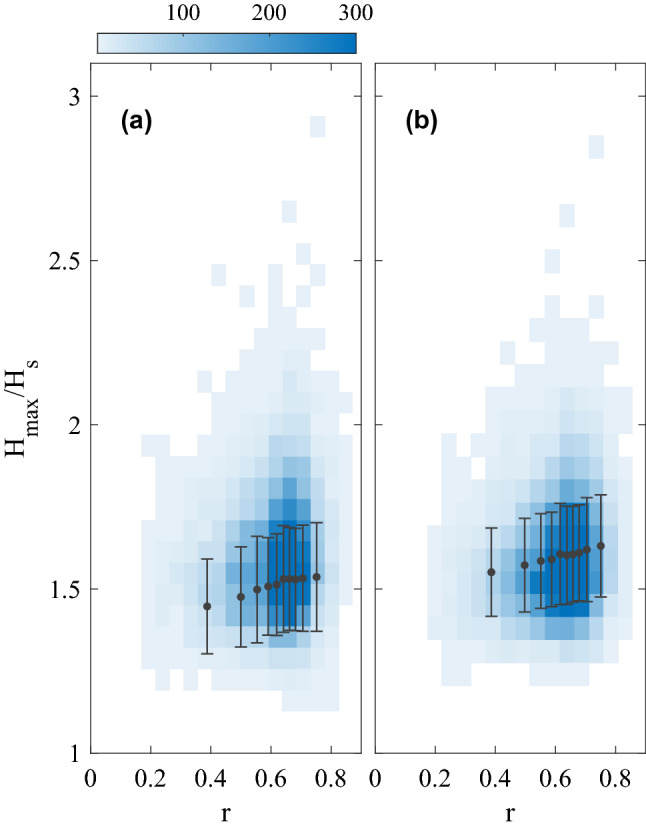
Maximum non-dimensional wave height $H/H_s$ within 30 min, as function of crest-trough correlation *r*. Individual data are shown as blue scatter plot, with data density given by the colour bar, and constant-size bin averages and $\pm 1\sigma$ are shown in black. $H_{max}$, $H_s$, and *r* are taken from (**a**) observations, (**b**) Monte-Carlo simulation.

**Figure 4:**
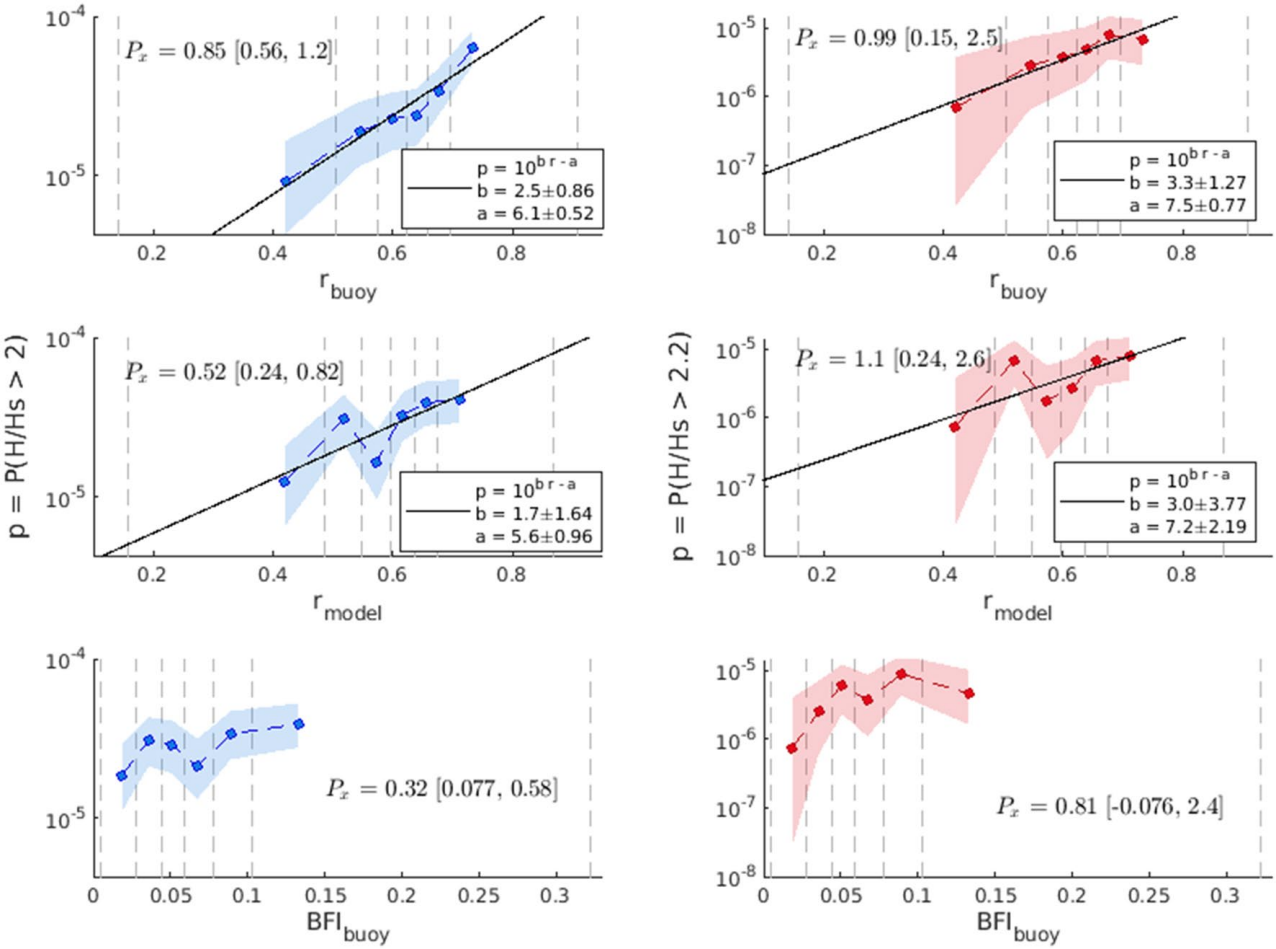
Rogue wave probability as functions of spectral wave parameters. Left column: Probabilities of normalized wave height >2.0, $p = P(H/H_s>2)$. Right column: Probabilities of normalized wave height >2.2, $p = P(H/H_s>2.2)$. Top row: variability with observed crest-trough correlation $r_{buoy}$. Middle row: variability with crest-trough correlation from the model $r_{model}$. Bottom row: variability with observed Benjamin Feir Index $BFI_{buoy}$. Shading indicates the 95% credible interval and vertical dashed lines indicate the edges of the parameter bins. Values for predictive power $P_x$ and best fit parameters are stated.

### Rogue wave occurrence rates

It is convenient to plot wave and crest height exceedance probabilities *P* as $\ln (-\ln (P))$ versus the normalized heights $\eta /H_s$, $H/H_s$ on a logarithmic scale (Fig. 2). On such a plot the Rayleigh distribution (Eq. 1) and distributions allowing for second order corrections fall on straight lines and any deviations are readily visible^8^. Our observations follow the standard model^13–15^ up to $H/H_s<2.1$ and $\eta /H_s<1.1$ but beyond that they show a dramatic increase of probabilities of high waves. As commonly observed, probabilities of wave heights $H/H_s$ exceeding a given value are lower than given by the Rayleigh distribution, due to the finite spectral width of ocean wave data^2^. Extrapolation of the observed distributions would give a probability of the November 17 rogue wave as 1 in $5\times 10^9$ and the rogue crest as 1 in $10^{14}$, several orders of magnitude less frequent than observed. In particular, the extreme crest height $\eta /H_s=1.98$ would be a nearly impossible event (1 in 31 million years) if based on the extrapolation of moderate waves.

**Figure 5:**
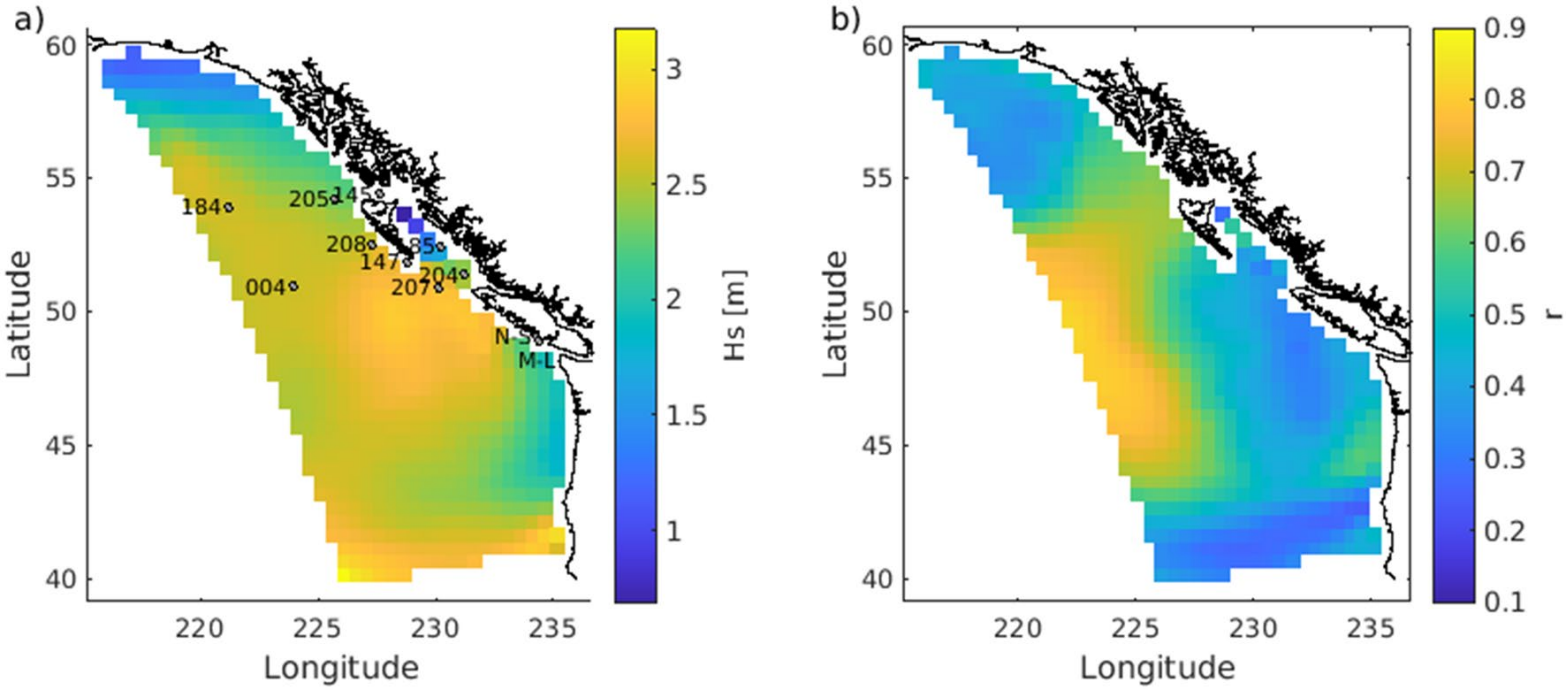
Example forecast output for March 22, 2020 00h UTC of (**a**) significant wave height $H_s$ and (**b**) crest-trough correlation *r*. These model output fields form the basis of a rogue wave risk prediction. Grey markers in (**a**) are locations of buoys utilized in this study. M-L and N-S marking the location of the coastal MarineLabs and Nearshore buoys, respectively and the numbered markers are the buoys from Fisheries and Oceans Canada where the complete names are the numbers preceded by a ‘C46’ prefix.

**Figure 6:**
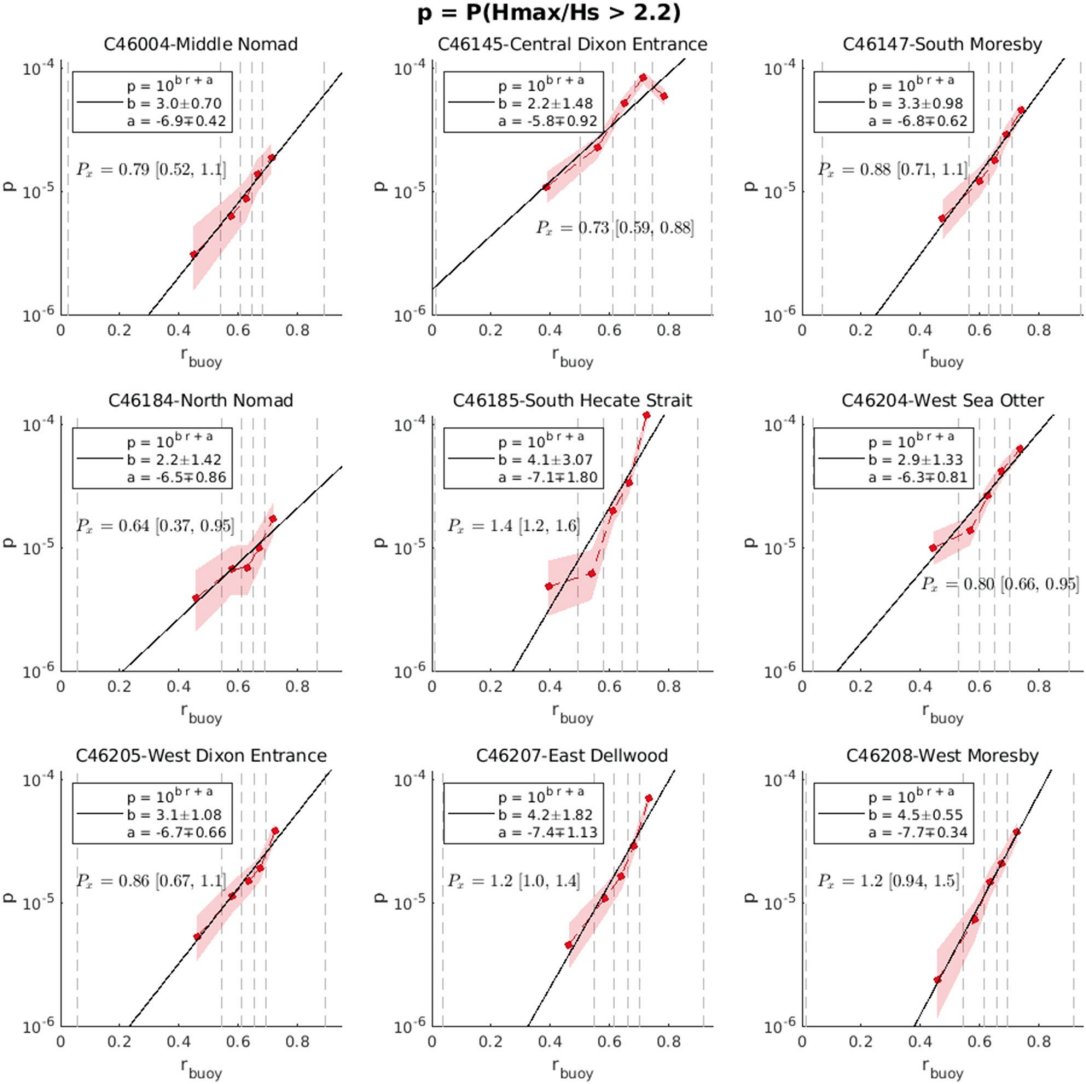
Variation of rogue wave probability $p=P(H_{max}/H_s>2.2)$ with observed crest-trough correlation $r_{buoy}$ from Fisheries and Oceans Canada buoys. Data sets include time series from 2010 to 2021 where quality control was performed and the record appears correct. Shading indicates the 95% credible interval and vertical dashed lines indicates the edges of the parameter bins.

To test whether the buoy observations are consistent with the linear mechanism of wave superposition allowing for the Stokes correction, we perform a Monte-Carlo simulation^8,30,31^ based on the observed wave spectra, and add bound harmonics up to fourth order^8^ (see “Methods”). This way we obtain a synthetic surface elevation record with the same characteristics as the one recorded in the ocean, but which does not include nonlinear wave interactions and therefore no modulational instability. The wave and crest heights from the simulations are in good agreement with the observed wave height distributions. In particular the rapid increase of rogue wave probabilities over the Rayleigh distribution or $\text{second}$ order corrections is well captured (Fig. 2). This supports random superposition of Stokes waves as the leading cause for rogue waves.

## Rogue wave risk prediction

The crest-trough correlation *r*^14,32^ (see “Methods”) is strongly correlated with the maximum normalized wave height in a record (Fig. 3). Observed and synthetic data show similar maximum wave heights for a given correlation value. The largest normalized wave heights are clearly associated with high correlation values (Fig. 3). A recent study found that in wave buoy observations the probability $P(H/H_s>2)$ increased by about one order of magnitude for the highest *r* value compared to records with the lowest *r*^25^. We performed a similar analysis to evaluate how the rogue wave probability *p* varies with sea state parameters *r* and BFI (see “Methods”). This analysis utilizes surface elevation measurements from the MarineLabs buoy and an additional nearby buoy, 2 km offshore in 20 m water depth referred to as the Nearshore buoy, resulting in a combined 28 months of data. Here, *p* is either defined as the probability of a wave exceeding $2H_s$ or $2.2H_s$.

Based on this observational data set *r* shows substantial correlation with *p* while BFI is practically ineffective (Fig. 4). The predictive power $P_x$ of *r* for $p = P(H/H_s>2)$ is 0.85 with a lower bound of 0.56, and *p* ranging from $9.1\times 10^{-6}$ to $6.3\times 10^{-5}$. That is, the occurrence rate of a rogue wave exceeding $2H_s$ varies from approximately 1 in 110,000 for the lowest *r* value to 1 in 16,000 during records with the highest r value. For $p = P(H/H_s>2.2)$, *r* has a higher predictive power of 0.99, however with a lower bound of 0.15, and *p* ranging from $7.2\times 10^{-7}$ to $6.9\times 10^{-6}$. The occurrence rate of a rogue wave exceeding $2.2H_s$ varies from 1 in 1,400,000 to 1 in 140,000. One of the issues that arises when analyzing extreme events is the limited amount of data, which has the unfortunate consequence of increasing the confidence bounds of the rogue wave probability exceeding $2.2H_s$ compared to $2H_s$. BFI has a predictive power of only 0.32 with lower bound of 0.077 for $p = P(H/H_s>2)$ and 0.81 for $p = P(H/H_s>2.2)$ with a lower bound of -0.07. The confidence bounds on $P_x$ indicate that BFI is not a robust prediction parameter and therefore would perform poorly as a rogue wave risk indicator.

The benefit of a spectrally derived parameter such as *r* relating to increased rogue wave risk, is that it can be obtained from standard spectral wave forecast models (Fig. 5). However, despite the regional WW3 model predicting $H_s$ with high reliability with a correlation coefficient of 0.96, the models prediction of *r* at the near shore location of the MarineLabs and Nearshore buoy is less reliable with a correlation coefficient of 0.61 and a scatter index of 15%. Even so, the crest-trough correlation from the model $r_{model}$ demonstrates a strong positive correlation with rogue wave probability. Additionally, $r_{model}$ has similar high predictive power for rogue wave probability ($p = P(H/H_s>2.2)$) as *r* obtained from the observations $r_{buoy}$.

Thus far the demonstration of the correlation between rogue wave probability and *r* has been restricted to coastal areas^25^ and it has been noted that the relationships between *p* and sea state parameters will likely depend on location^33^. Long term surface elevation records, which are the basis of the analysis presented here, and in^25^ are few. However, time series of hourly wave height maxima $H_{max}$ and significant wave height $H_s$ are more readily available. Using these bulk statistics a similar analysis can be performed using longterm time series from buoys available through the Marine Environmental Data Section (MEDS) of Fisheries and Ocean Canada (DFO)^34^. Records are available for a wide range of offshore buoy locations (Fig. 5) and thus the rogue wave prediction can be calibrated for the full domain of the operational wave forecast (Fig. 6). The caveat with this analysis is it does not register multiple rogue wave events in a given record segment and the number of waves in a record is estimated from the mean period. The models forecast of *r* also improves at offshore locations. For example, at an open ocean buoy (C46004) the correlation coefficient increased to 0.78 and the scatter index decreased to 10%.

To formalize the risk prediction forecast we took the average of the semi logarithmic fits from the Marinelabs and Nearshore data set and 9 DFO buoys to get the following equations relating rogue wave probability and *r*.

2 $$\begin{aligned} P\left( H/H_s>2 \right) = 10^{(2.54\pm 0.48)r-(5.70\pm 0.29)} ,\text { and}\,\, P\left( H/H_s>2.2 \right) = 10^{(3.31\pm 0.86)r-(6.90\pm 0.53)} \end{aligned}$$

We didn’t find substantial differences in the slope of the fits when grouped by depth, region or distance from the coast. The spatial and temporal fields of *r* can be output from the wave model and using the above equations the probability of extreme wave events can be calculated. This analysis was only performed for extreme wave heights as wave crests by definition are not affected by crest-trough correlation.

## Discussion

The spectrogram of the surface elevation (Fig. 1b) highlights the rogue wave as an event where a wide range of energetic spectral wave components align. These components are steep compared to the background wave field with enhanced higher order Stokes corrections, and their superposition generates extreme crest heights. The Stokes enhancement of less energetic, and therefore less steep waves is much weaker (see Eq. 3) and their superposition results in moderate crest heights, only. This explains the dramatic increase of rogue wave probabilities for $H/H_s\gtrsim 2.1$ which is not captured by second-order Stokes corrections.

To develop a rogue wave risk prediction system we evaluated how the rogue wave probability *p* changes with certain sea state parameters. Based on the strong correlation with rogue wave probability at offshore and near-shore locations, we present crest-trough correlation *r* to be used for rogue wave risk prediction in wave forecasts. *r* is a logical parameter to be linked with the generation of rogue waves as it is an estimate of the auto-correlation between the crest heights and the trough depths. Therefore, in narrow-banded seas the crests and successive troughs are approximately the same size resulting in high *r* values. Narrow-banded seas also maintain wave groups and rogue waves are more likely to form in ‘groupy’ sea states. Additionally, *r* can be easily computed by a routine wave forecast as demonstrated in Fig. 5 and alongside a forecast for $H_s$ provides a complete risk assessment where overlapping areas of high $H_s$ and *r* pose the greatest risk.

## Methods

### Linear rogue wave simulation

Long time series of high-resolution wave observations under natural conditions are still rare but are required for assessing the occurrence rate of rogue waves. Many height distributions are evaluated with observed probabilities $\le 10^{-5\,\,}$^13,35^ and only a few studies reach $P\le 10^{-7}\,\,$^25,27,29,36^. Alternatively, synthetic time series can provide good statistics on low-probability events and have the advantage to test non-linear (up to a desired order) or linear dynamics. Here we use the Matlab toolbox WAFO^37^ to synthesize surface elevation from linear superposition of spectral components with sine and cosine terms that are independent and Gaussian^30^. For each of the 11,600 data segments from the buoy a thirty-minute synthetic surface elevation $h_{lin}$ with 5Hz resolution and the same spectral characteristics as the observations is generated. All individual waves, defined as data between consecutive zero-down crossings, are then modified with fourth order Stokes correction^12^

3 $$\begin{aligned} h_4=a \cos (kx)+ \left( \frac{1}{2}\varepsilon +\frac{17}{24}\varepsilon ^3\right) a\cos (2kx)+\frac{3}{8}\varepsilon ^2 a\cos (3kx)+\frac{1}{8}\varepsilon ^3 a\cos (4kx) \end{aligned}$$

with wave amplitude *a*, wave steepness $\varepsilon =ak$, and the wavenumber *k* is obtained iteratively from the dispersion relation $\omega ^2=gk\tanh (kD)$, with $\omega =2\pi /T$, zero-down crossing wave period *T*, gravitational acceleration *g* and water depth *D*. The first term on the r.h.s. of Eq. (3) represents the linear component $h_{lin}$, the remaining terms the Stokes correction. For each data segment the significant wave height $H_s=4\sigma$, where $\sigma$ is the standard deviation of the surface elevation, the crest-trough correlation *r* and BFI (see below) are calculated. For the entire observed and synthetic time series all individual crest heights and trough-to-crest wave heights are extracted and normalized by $H_s$ of the segment.

### Wave model

A WAVEWATCHIII^®^ (WW3) regional wave model was calibrated for the Northeast Pacific where the domain extends from northwest and southwest corners located at approximately ($60^{\circ }$ N, $145^{\circ }$ W) and ($40^{\circ }$ N, $134^{\circ }$ W) to the coast (Fig. 5). The model uses a triangular unstructured grid with variable resolution of 1000 m nearshore to 5 km offshore. An implicit solving scheme was used in this study with global time step of 600 s. The model resolves 36 frequency bins from 0.035 to 0.98 Hz and 36 direction bins spaced every 10. Boundary conditions are imposed at the western and southern open boundary nodes extracted from the 0.25 Global Deterministic Wave Prediction System (GDWPS) every 5 km (https://weather.gc.ca/model_forecast/wave_e.html). The model is forced with 2.5 km resolution winds from High Resolution Deterministic Prediction System (HRDPS). The ST4 package was used to parameterize wind input and dissipation with growth parameter ^38^. In addition, nonlinear four wave interaction using discrete interaction approximation, DIA (NL1)^39^, JONSWAP bottom friction (BT1), depth-limited breaking^40^ (DB1), triad interactions (TR1)^41^, and a linear input term (LN1) have been used for the computations. To produce the low resolution *r* field in Fig. 5 the WW3 model was run and point spectra were output every . *r* was then calculated from Eq. (7).

### Rogue wave predictors

To assess the nonlinear mechanism of rogue wave generation by the modulation instability we use the Benjamin Feir Index (BFI)^17^. BFI is calculated as the ratio of wave steepness $\varepsilon$ to spectral bandwidth $\nu$^42^.

4 $$\begin{aligned} \text {Benjamin Feir Index, } \mathrm {BFI}&= \sqrt{2}\left( \frac{\varepsilon }{\nu }\right) \end{aligned}$$

5 $$\begin{aligned} \text {Wave Steepness, } \varepsilon&= \sqrt{k_p^2 m_0} \end{aligned}$$

6 $$\begin{aligned} \text {Spectral Bandwidth, } \nu&= \sqrt{\frac{m_0m_2}{m_1^2}-1} \end{aligned}$$

where $k_p$ is the wavenumber associated with the peak period $T_p$ calculated from the weighted spectrum^43^, and $m_n = \int _{0}^{\infty } f^n S(f)df$ is the $n^{th}$ spectral moment.

The crest-trough correlation *r* has been suggested as a parameter to relate to wave height distributions^14^. Crest-trough correlation *r* is the auto-correlation of the sea surface elevation at half the wave period. It can be estimated from the spectral density, *S*(*f*) using the Wiener-Khinchin theorem. Following^15^ we compute *r* as,

7 $$\begin{aligned} r = \frac{1}{m_0}\sqrt{\rho ^2+\lambda ^2}, \text { where}\,\,\, \rho = \int _{0}^{\infty } S(f)\cos (2 \pi f \tau )df \text { and}\,\,\, \lambda = \int _{0}^{\infty } S(f)\sin (2 \pi f \tau )df, \end{aligned}$$

where $\tau = \frac{\bar{T}}{2}$ is the lag time at half the spectral mean period $\bar{T} = \frac{m_0}{m_1}$.

### Estimation of rogue wave probabilities

We evaluate the univariate rogue wave probability *p* with uncertainties following the same method as Häfner et al. (2021) outlined briefly below^25^. We define *p* as the probability that the next wave height will exceed the threshold $2H_s$ or $2.2H_s$. To evaluate how *p* varies with a wave parameter *x* we split *x* and $H/H_s$ into *N* bins with approximately equal number of observations in each bin. We assume that the number of rogue waves $n^+$ and the number of non-rogue waves $n^-$ in each bin are identically and independently distributed (iid) according to a binomial distribution with probability *p*. For the prior distribution of *p* we assume a Beta distribution with parameters $\alpha _0 = 1$ and $\beta _0 = 3000$ for $P(H/H_s>2)$ and $\beta _0 = 16{,}000$ for $P(H/H_s>2.2)$ from the Rayleigh distribution. Applying Bayes theorem we find the posterior probability for *p* is another Beta distribution, since the Beta prior for *p* is conjugate to the binomial distribution of $n^+$.

8 $$\begin{aligned} P(p|n^+,n^-) = \mathrm {Beta}(n^++\alpha _0, n^-+\beta _0) \end{aligned}$$

Our estimate of *p* with uncertainties is calculated from the median and the 95% credible interval of Eq. (8) (based on 2.5th and 97.5th percentiles). The benefit of this analysis is to be able to generate uncertainties for *p* to determine whether the variation with *x* is meaningful. Here are a few things to note. Firstly, the purpose of the Beta prior is only to constrain *p* to a reasonable order of magnitude so the exact choice of $\alpha _0$ and $\beta _0$ does not influence final results. Secondly, this analysis hinges on the assumptions that *p* is iid distributed in each bin, which isn’t the case if *p* depends on more than one parameter. Therefore, these uncertainties indicate the level of confidence in our best estimate of *p* if we only consider one parameter at a time.

For buoys where surface elevation is unavailable, the normalized wave is given by $H_{max}/H_s$ where $H_{max}$ is the maximum wave in a record and the total number of waves $N_0$ is estimated using the mean period $\bar{T}$ and the record length *RL* where, $N_0 = RL/\bar{T}$. To evaluate if this simplifying approach is acceptable we perform these simplifications on our Nearshore and MarineLabs data set to compare with results using the full sea surface elevation. Using $H_{max}/H_s$ excludes only three rogue events in the data set. Those events correspond to segments where there were two waves with height exceeding $2H_s$. Overall, there is no appreciable difference using $H_{max}$ and *T*_01_ to get *N*_0_, however there is the limitation that the limit of rogue waves flagged in a record is one.

To evaluate the degree of variability of *p* with parameter *x* we use the predictive power defined as, where $i_{max}$ is the bin index where *x* is highest and is bin index where *x* is lowest. This measures how much *p* varies with *x* if we only consider this one parameter. The uncertainty in is quantified through Monte Carlo sampling, based on the known distributions of $p_{imax}$ and given in Eq. (8) and the 95% confidence interval calculated from the 2.5th and 97.5th percentiles of the distribution of .

### MEDS buoys

The offshore buoys data sets are provided by the Marine Environmental Data Section (MEDS) buoys of Fisheries and Oceans Canada (DFO) (https://meds-sdmm.dfo-mpo.gc.ca)^34^. We utilized the long term time series of $H_{max}$, $H_s$ and frequency spectra. The open ocean buoys (C46004 and C46184) are 6m NOMAD buoys, the rest are AXYS 3m discus buoys. They record vertical acceleration at 1 Hz sampling rate for 34 minutes every hour and output bulk wave statistics. This work only used data which had received a quality control check and the record appears correct from 01/01/2010 to 15/08/2021. Any other suspicious data sections were removed. Due to outages in service and erroneous records the time series from the MEDS buoys are variable in length and do not necessarily span the full 11 year. The buoys location are found in Fig. 5a.

## Data Availability

The wave model data and the MarineLabs buoy data are available from the corresponding author on reasonable request. Data access is restricted to research and educational applications. Data from the Fisheries and Oceans Canada buoys can be accessed at https://meds-sdmm.dfo-mpo.gc.ca.

## Code availability

The generation of the synthetic surface elevation data was performed with the free Matlab toolbox “WAFO”^37^. WAVEWATCH III ^®^ is open source code^28^.
